# Assessment of Concordant Human Papillomavirus Infection With 9-Valent Vaccine Types Across Anogenital Sites in Young Men

**DOI:** 10.1093/infdis/jiaf564

**Published:** 2025-12-16

**Authors:** Cara J Broshkevitch, Anna R Giuliano, Joel M Palefsky, Georges Nahhas, Stephen E Goldstone, Brady Dubin, Alfred Saah, Alain Luxembourg, Christine Velicer, Joseph E Tota

**Affiliations:** Department of Epidemiology, Biostatistics and Research Decision Sciences, Merck Research Laboratories, Merck & Co., Inc., Rahway, New Jersey, USA; Center for Immunization and Infection Research in Cancer (CIIRC), Moffitt Cancer Center and Research Institute, Tampa, Florida, USA; Department of Medicine, University of California San Francisco, San Francisco, California, USA; Department of Epidemiology, Biostatistics and Research Decision Sciences, Merck Research Laboratories, Merck & Co., Inc., Rahway, New Jersey, USA; Department of Surgery, Icahn School of Medicine at Mount Sinai, New York, New York, USA; Merck Research Laboratories, Merck & Co., Inc., Rahway, New Jersey, USA; Global Medical and Scientific Affairs, Merck Research Laboratories, Merck & Co., Inc., Rahway, New Jersey, USA; Vaccine Clinical Development, Merck Research Laboratories, Merck & Co., Inc., Rahway, New Jersey, USA; Department of Epidemiology, Biostatistics and Research Decision Sciences, Merck Research Laboratories, Merck & Co., Inc., Rahway, New Jersey, USA; Department of Epidemiology, Biostatistics and Research Decision Sciences, Merck Research Laboratories, Merck & Co., Inc., Rahway, New Jersey, USA

**Keywords:** concordant infection, human papillomavirus, HPV infection, HPV vaccines, males

## Abstract

**Background:**

The global prevalence of and risk factors associated with same-type HPV infection across multiple anogenital sites among men has not been quantified.

**Methods:**

Men aged 16–27 years participating in a multi-country 4-valent HPV vaccine trial (NCT00090285) were assessed at baseline for prevalent HPV infection at penile/scrotal and perineal/perianal sites (heterosexual men [HM] and men who have sex with men [MSM]) and additionally at intra-anal sites (MSM). Concordant infection with 9-valent HPV (9vHPV) vaccine types (6/11/16/18/31/33/45/52/58) was defined as same-type 9vHPV infection at 2 or 3 sites.

**Results:**

3363 HM and 595 MSM were included. Prevalence of concordant 9vHPV infection at 2 anogenital sites was 3.7% among HM and 9.1% among MSM, and 8.2% at 3 sites (including the intra-anal site) among MSM. HPV6 and HPV16 were most likely to occur at multiple anogenital sites in HM and MSM, with strong agreement observed between perineal/perianal and anal sites among MSM for HPV6 (Cohen's kappa, 0.78; 95% CI, .69–.87) and HPV16 (0.61; 95% CI, .50–.73).

**Conclusions:**

Concordant anogenital 9vHPV infection was more common among MSM than HM, which is consistent with evidence that MSM are at increased risk of HPV-related cancer across multiple anogenital sites.

**Clinical Trial Registration:**  ClinicalTrials.gov, NCT00090285

## Teaser

Concordant anogenital HPV infection was more common in men who have sex with men than heterosexual men; HPV6 and HPV16 were most commonly detected

HPV vaccination may protect against initial HPV infection and acquisition of infections at other anogenital sites

Human papillomavirus (HPV) infection at anogenital sites is common and causes approximately 50% of penile cancers and over 80% of anal cancers in men [1–3]. Men infected with HPV may have concordant infection, defined as same-type HPV infection at 2 or more anatomical sites. However, data are limited on the global prevalence of concordant anogenital HPV infection in men.

Previous studies assessing concordant anogenital infection with high-risk HPV types were limited to men attending a sexually transmitted infection (STI)/HIV clinic in a single country, or to unvaccinated men who have sex with men (MSM) with HIV [4, 5]. These studies reported 7%–11% prevalence of concordant infection at penile and anal sites, with the highest prevalence of concordant infection at anal and perianal sites among MSM with HIV [4, 5]. However, neither study assessed factors associated with concordant HPV infection at multiple anogenital sites. It is important to evaluate concordance of anogenital HPV infection among men globally, and to compare prevalence and factors associated with concordance among HM and MSM. Data on concordance of anogenital HPV infection may be used to further characterize the burden of HPV infection among infected men and to better understand the potential benefit of HPV vaccination.

In this analysis, we used data from a multi-country 4vHPV vaccine trial (V501-020) enrolling young men to assess the prevalence of concordant HPV infection with 9-valent HPV (9vHPV) vaccine types across anogenital sites among heterosexual men (HM) and MSM, as well as factors associated with concordant anogenital 9vHPV infection.

## METHODS

### Data Source

Data in this cross-sectional analysis were from HM (*N* = 3363) and MSM (*N* = 595) enrolled in the V501-020 trial; NCT00090285 [6]. Details of the trial have been previously reported [6]. The study enrolled men living in Australia, Brazil, Canada, Costa Rica, Croatia, Finland, Germany, Mexico, the Netherlands, Norway, the Philippines, Peru, Portugal, South Africa, Spain, Sweden, Taiwan, and the United States; MSM residing in South Africa were not included in the study. Briefly, key inclusion criteria of the V501-020 trial were HM (aged 16–23 years) who had 1–5 lifetime exclusively female sex partners, or MSM (16–26 years) with 1–5 lifetime sex partners who reported engaging in either insertive or receptive anal intercourse, or oral sex with a male partner, within the past year [6]. HM and MSM with clinical evidence or history of anogenital warts or dysplasia, or genital lesions at screening that were suggestive of an infection with non-HPV sexually transmitted disease were excluded from the V501-020 trial [6]. HM and MSM diagnosed with HIV prior to the first day of the study were excluded. During follow-up, HM from South Africa and all MSM were tested for HIV annually, whereas HM from other countries were only tested if clinically indicated [6].

The V501-020 trial was sponsored by Merck Sharp & Dohme LLC, a subsidiary of Merck & Co., Inc., Rahway, NJ, USA, and was conducted in accordance with principles of Good Clinical Practice and was approved by the appropriate institutional review boards and regulatory agencies. All participants (or their legally authorized representatives) provided written informed consent at the start of the study.

### HPV DNA Sampling and Assessment

Penile, scrotal, and perineal/perianal specimens were obtained from all study participants (HM and MSM) at baseline; intra-anal specimens were collected from MSM participants only. All specimens were HPV genotyped using multiplex polymerase chain reaction (PCR)-based assays [7, 8] to identify DNA for the 9 HPV types (6, 11, 16, 18, 31, 33, 45, 52, and 58) targeted by the 9-valent HPV (9vHPV) vaccine.

### Type-specific HPV Serologic Assessment

Competitive Luminex immunoassay (cLIA) was used to identify antibodies from patient serum against individual 9vHPV vaccine types at baseline [9]. Seropositivity for an HPV type was defined as an HPV type-specific value at or above the serostatus cutoff for that HPV type, and seronegativity was defined as a value below the serostatus cutoff for that HPV type. Serostatus cutoffs (milli-Merck units per milliliter [mMU/mL]) for specific 9vHPV types were as follows: 30 (HPV6), 16 (HPV11), 20 (HPV16), 24 (HPV18), 10 (HPV31), and 8 (HPV types 33, 45, 52, and 58) [10, 11].

### Analysis Population and Assessments

The analysis population included all participants randomly allocated to the placebo or vaccine arms who underwent testing for 9vHPV types and had a valid PCR result at baseline from swabs at each anogenital site. Participants with missing PCR results at baseline were not included in the analysis population.

A concordant 9vHPV infection was defined as same-type 9vHPV infection at 2 or more anogenital sites. Participants with HPV infections that did not meet the concordant infection definition, including different type infections at the same site and different type infections at different sites, were considered to have non-concordant infection.

### Statistical Analysis

The association between baseline characteristics and risk of any concordant 9vHPV infection (ie, same-type infection with ≥1 9vHPV type) at anogenital sites was assessed using logistic regression, separately for HM and MSM. Age-adjusted odds ratios (ORs) and 95% CIs were reported.

To assess the degree of 9vHPV infection concordance between anogenital sites, Cohen's kappa was used to quantify concordance at pairs of sites. Fleiss's kappa was used to evaluate concordance across all 3 sites among MSM. Concordance was quantified for any 9vHPV type, as well as for HPV types 6 and 16 individually, which are the most prevalent HPV types identified in anogenital warts (HPV6) and HPV-related cancers/precancers (HPV16) in men [12–14].

All analyses were conducted using SAS version 9.4 (SAS Institute Inc., Cary, NC, USA).

## RESULTS

### Demographic and Behavioral Characteristics of HM and MSM

HM were younger on average than MSM, with over half of HM aged 16–20 years (55.2%), while most MSM were aged 21–27 years (70.8%) (Table 1). A large proportion of HM was enrolled from Latin America (41.6%), while MSM commonly resided in North America (43.0%). Similar proportions of HM and MSM were current tobacco users (36.5% vs 40.2%). Most men reported 0–3 lifetime sex partners (HM, 66.7%; MSM, 49.8%), although many MSM reported 4–5 lifetime male sex partners (45.9%), including 3–5 lifetime partners with insertive (38.0%) and receptive (39.0%) anal intercourse. Lifetime condom use was frequent (‘always' or “more than half the time’) in both HM (69.2%) and MSM (80.5%), and most men were not circumcised (HM, 63.3%; MSM, 55.8%).

**Table 1.** Baseline Demographic and Behavioral Characteristics of the Study Population

*N* (%)	HM (*N* = 3363)	MSM (*N* = 595)
**Age^a^**		
16–20	1855 (55.2)	174 (29.2)
21–27	1508 (44.8)	421 (70.8)
**Geographic region**		
North America	790 (23.5)	256 (43.0)
Latin America	1400 (41.6)	132 (22.2)
Europe	366 (10.9)	122 (20.5)
Asia-Pacific	271 (8.1)	85 (14.3)
Africa	536 (15.9)	N/A
**Tobacco use at baseline**		
Never used	1919 (57.1)	301 (50.6)
Ex-users	217 (6.5)	55 (9.2)
Current user	1227 (36.5)	239 (40.2)
**Age at first intercourse^b^**		
< 15	418 (12.4)	67 (11.3)
15–19	2673 (79.5)	393 (66.1)
≥ 20	266 (7.9)	115 (19.3)
**Number of lifetime male sex partners^b^**		
0–3	N/A	296 (49.8)
4–5	N/A	273 (45.9)
**Number of lifetime female sex partners^b^**		
0–3	2243 (66.7)	146 (24.5)
4–5	1112 (33.1)	2 (0.3)
**Number of lifetime partners with insertive anal intercourse^b^**		
0	N/A	70 (11.8)
1	N/A	141 (23.7)
2	N/A	131 (22.0)
3–5	N/A	226 (38.0)
**Number of lifetime partners with receptive anal intercourse^b^**		
0	N/A	59 (9.9)
1	N/A	130 (21.9)
2	N/A	147 (24.7)
3–5	N/A	232 (39.0)
**Number of new male partners in last 6 m^b^**		
0	N/A	212 (35.6)
1	N/A	229 (38.5)
≥ 2	N/A	129 (21.7)
**Number of new female partners in last 6 m^b^**		
0	2005 (59.6)	132 (22.2)
1	1089 (32.4)	11 (1.9)
≥ 2	262 (7.8)	3 (0.5)
**Frequency of lifetime condom use^b^**		
Always	1237 (36.8)	249 (41.9)
More than half the time	1090 (32.4)	230 (38.7)
Less than half the time	690 (20.5)	60 (10.1)
Never	338 (10.1)	32 (5.4)
**Frequency of condom use in the last 6 m**		
Always	1184 (35.2)	253 (42.5)
More than half of the time	670 (19.9)	106 (17.8)
Less than half of the time	505 (15.0)	46 (7.7)
Never	929 (27.6)	162 (27.2)
**Circumcision**		
No	2128 (63.3)	332 (55.8)
Yes	1235 (36.7)	263 (44.2)

Abbreviations: HM, heterosexual men; MSM, men who have sex with men.

^a^The inclusion criterion for age was 16–26 y; however, some men aged 26 y at screening subsequently turned 27 y of age by the enrollment visit.

^b^Categories have missing values for MSM.

### Prevalence of Concordant 9vHPV Anogenital Infection Among HM and MSM

Among HM, the overall prevalence at baseline of any 9vHPV infection was 9.8% at 1 site (penile/scrotal or perineal/perianal) and 3.7% at 2 sites (penile/scrotal and perineal/perianal). The overall prevalence of any 9vHPV infection among MSM was 14.8% at 1 site, 15.5% at 2 sites (penile/scrotal, perineal/perianal, or intra-anal), and 8.2% at 3 anogenital sites (penile/scrotal, perineal/perianal, and intra-anal sites).

Among HM, the overall prevalence of 9vHPV concordant infection at 2 anogenital sites (penile/scrotal site and perineal/perianal site) was 3.7% (Table 2). Prevalence rates of concordant infection by individual 9vHPV types in HM were highest for HPV6 (0.7%), HPV16 (0.7%), HPV18 (0.6%), and HPV52 (0.6%). Among MSM, the overall prevalence of 9vHPV concordant infection at 2 anogenital sites (penile/scrotal and perineal/perianal) and 3 anogenital sites (penile/scrotal, perineal/perianal, and intra-anal sites) were 9.1% and 8.2%, respectively. Prevalence rates of concordant infection by individual 9vHPV types in MSM were highest for HPV6 (2.9%), HPV16 (2.0%), and HPV18 (1.8%) for concordant infection at 2 anogenital sites, and HPV6 (2.5%), HPV16 (1.8%), and HPV18 (1.5%) for infection at 3 sites.

**Table 2.** Concordant 9vHPV Anogenital Infection Status at Baseline Among Heterosexual Men and Men Who Have Sex With Men

HPV Type,	HM (*N* = 3363)^a^			MSM (*N* = 595)^b^			
	HPV Negative at All Sites^a^ *n* (%)	Non-concordant Infection^a^ n (%)	Concordant Infection at 2 Sites^b^ *n* (%)	HPV Negative at All Sites^c^ *n* (%)	Non-concordant Infection^d^ *n* (%)	Concordant Infection at 2 Sites^d^ *n* (%)	Concordant Infection at 3 Sites^e^ *n* (%)
6	3257 (96.9)	83 (2.5)	23 (0.7)	539 (90.6)	39 (6.6)	17 (2.9)	15 (2.5)
11	3348 (99.6)	13 (0.4)	2 (0.1)	567 (95.3)	23 (3.9)	5 (0.8)	3 (0.5)
16	3242 (96.4)	98 (2.9)	23 (0.7)	549 (92.3)	34 (5.7)	12 (2.0)	11 (1.8)
18	3299 (98.1)	43 (1.3)	21 (0.6)	561 (94.3)	23 (3.9)	11 (1.8)	9 (1.5)
31	3311 (98.5)	39 (1.2)	13 (0.4)	569 (95.6)	21 (3.5)	5 (0.8)	5 (0.8)
33	3341 (99.4)	13 (0.4)	9 (0.3)	583 (98.0)	11 (1.8)	1 (0.2)	1 (0.2)
45	3319 (98.7)	29 (0.9)	15 (0.4)	565 (95.0)	22 (3.7)	8 (1.3)	5 (0.8)
52	3266 (97.1)	77 (2.3)	20 (0.6)	576 (96.8)	17 (2.9)	2 (0.3)	1 (0.2)
58	3308 (98.4)	37 (1.1)	18 (0.5)	580 (97.5)	11 (1.8)	4 (0.7)	4 (0.7)
6/11/16/18/31/33/45/52/58	2908 (86.5)	329 (9.8)	126 (3.7)	422 (70.9)	119 (20.0)	54 (9.1)	49 (8.2)

Abbreviations: HM, heterosexual men; HPV, human papillomavirus; MSM, men who have sex with men; PCR, polymerase chain reaction.

“*N*” indicates the number of HM or MSM randomly assigned to either the placebo or vaccine arm with valid baseline PCR results for swabs at all anogenital sites among participants having all 9vHPV types tested.

“*n*” indicates the number of HM or MSM with 9vHPV infection detected at baseline.

^a^Anogenital sites for HM comprise penile/scrotal or perineal/perianal sites.

^b^Anogenital sites for HM comprise penile/scrotal and perineal/perianal sites.

^c^Anogenital sites for MSM comprise penile/scrotal, perineal/perianal, or intra-anal sites.

^d^Anogenital sites for MSM comprise penile/scrotal and perineal/perianal sites.

^e^Anogenital sites comprise penile/scrotal, perineal/perianal, and intra-anal sites.

Cohen's kappa values showed fair-to-moderate agreement in same-type 9vHPV DNA detection across penile/scrotal and perineal/perianal sites among HM (Cohen's kappa, 0.45; 95% CI, .40–.50) and MSM (0.35; 95% CI, .26–.45), and fair agreement across penile/scrotal and anal sites among MSM (0.24; 95% CI, .16–.32) (Table 3). In contrast, strong agreement was observed across perineal/perianal and anal sites among MSM for any 9vHPV type (Cohen's kappa, 0.73; 95% CI, .67–.80), HPV6 (0.78; 95% CI, .69–.87), and HPV16 (0.61; 95% CI, .50–.73). Among MSM, the Fleiss’ kappa value for agreement in same-type 9vHPV DNA detection across all 3 anogenital sites (penile/scrotal, perineal/perianal, and intra-anal) was 0.46 (95% CI, .40–.52).

**Table 3.** **Agreement of Same-Type 9vHPV DNA Detection in Heterosexual Men and Men Who Have Sex With Men by Anogenital Site Using Cohen**’**s Kappa Values for Site Pairs**

Site Pair	Cohen's Kappa (95% CI)	
	HM^a^	MSM^b^
**Penile/scrotal – Perineal/perianal**	…	…
Any 9vHPV	.45 (.40–.50)	.35 (.26–.45)
HPV6	.41 (.30–.53)	.46 (.31–.61)
HPV16	.38 (.27–.49)	.39 (.22–.56)
**Penile/scrotal – Intra-anal**	…	…
Any 9vHPV	N/A	.24 (0.16–0.32)
HPV6	N/A	.30 (.17–.43)
HPV16	N/A	.24 (.12–.36)
**Perineal/perianal—Intra-anal**	…	…
Any 9vHPV	N/A	.73 (.67–.80)
HPV6	N/A	.78 (.69–.87)
HPV16	N/A	.61 (.50–.73)

Abbreviations: HM, heterosexual men; HPV, human papillomavirus; MSM, men who have sex with men; PCR, polymerase chain reaction.

One participant aged 15 y was excluded.

^a^Site pairs for HM include penile/scrotal and perineal/perianal concordant infections of same-type 9vHPV, based on PCR assay of swab specimens at baseline.

^b^Site pairs for MSM include penile/scrotal and perineal/perianal, penile/scrotal and anal, and perineal/perianal and anal concordant infections of same-type 9vHPV, based on PCR of swab specimens at baseline.

Among HM, the prevalence of non-concordant 9vHPV infection with a single HPV type at 1 anogenital site was 8.2%. The prevalence of infection with 2 or more non-concordant HPV types was 1.6% at 1 site and 0.5% at 2 sites (Supplementary Table 1). Among MSM, the prevalence of 9vHPV infection with a single HPV type at 1 anogenital site was 11.4%. The prevalence of 9vHPV infection with 2 or more non-concordant HPV types was 3.4% at 1 site, 4.5% at 2 sites, and 0.7% at 3 sites (Supplementary Table 1).

### Characteristics Associated With Concordant 9vHPV Infection Across Multiple Anogenital Sites Among HM and MSM

In age-adjusted analyses of concordant infection at 2 anogenital sites among HM, residence in Latin America (OR, 1.75; 95% CI, 1.02–3.01), Europe (OR, 2.19; 95% CI, 1.12–4.26), or Africa (OR, 2.55; 95% CI, 1.41–4.60) was associated with concordant 9vHPV infection (ie, same-type infection with ≥1 9vHPV type) relative to participants located in North America (Table 4). Factors related to sexual activity among HM, including younger age at first intercourse (15–19 vs ≥ 20 years: OR, 3.17; 95% CI, 1.14–8.77; < 15 vs ≥ 20 years: OR, 5.80; 95% CI, 1.96–17.10) and a higher number of lifetime female sex partners (4–5 vs 0–3: OR, 1.57; 95% CI, 1.10–2.26) were also associated with concordant 9vHPV infection. Current tobacco use (current vs never; OR, 1.44; 95% CI, 1.00–2.09) and seropositivity for HPV11 (vs seronegative or no serology; OR, 4.01; 95% CI, 1.62–9.94), HPV31 (OR, 4.42; 95% CI, 1.76–11.10), HPV45 (OR, 12.40; 95% CI, 1.11–13.90), HPV52 (OR, 3.72; 95% CI, 1.07–12.90) and HPV58 (OR, 3.49; 95% CI, 1.59–7.65) were also associated with concordant infection. In contrast, circumcision was associated with decreased odds of concordant 9vHPV infection after adjusting for age (yes vs no; OR, 0.60; 95% CI, .40–.90). In a multivariable logistic regression analysis of concordant 9vHPV infections, no factors were associated with concordant 9vHPV infection among HM, except for circumcision which was associated with decreased odds of concordant 9vHPV infection (yes vs no; OR, 0.49; 95% CI, .28–.85) among HM (Supplementary Table 2).

**Table 4.** Factors Associated With Concordant HPV Infection (Any 9vHPV Type) Across Multiple Anogenital Sites (Including Intra-Anal Sites) Among Heterosexual Men and Men Who Have Sex With Men

	HM (*N* = 3363)^a^			MSM (*N* = 595)^a^		
	*n* ^b^ (%)	Concordant 9vHPV Infection at 2 Sites, *n*^c^ (%)	Age-Adjusted OR (95% CI)	*n* ^b^ (%)	Concordant 9vHPV Infection at ≥2 Sites, *n*^c^ (%)	Age-Adjusted OR (95% CI)
**Age**						
21–27 y	1508 (44.84)	62 (4.11)	1.0	421 (70.76)	102 (24.22)	1.0
16–20 y	1855 (55.16)	64 (3.45)	.83 (.58–1.19)	174 (29.24)	39 (22.41)	.90 (.59–1.38)
**Geographic region**						
North America	790 (23.49)	18 (2.28)	1.0	256 (43.03)	35 (13.67)	1.0
Latin America	1400 (41.63)	56 (4.00)	1.75 (1.02–3.01)	132 (22.18)	43 (32.58)	3.04 (1.83–5.07)
Europe	366 (10.88)	18 (4.92)	2.19 (1.12–4.26)	122 (20.50)	45 (36.89)	3.78 (2.25–6.35)
Asia-Pacific	271 (8.06)	2 (0.74)	.29 (.07–1.26)	85 (14.29)	18 (21.18)	1.71 (.91–3.21)
Africa	536 (15.94)	32 (5.97)	2.55 (1.41–4.60)	N/A	N/A	N/A
**Tobacco use at baseline**						
Never used	1919 (57.06)	61 (3.18)	1.0	301 (50.59)	62 (20.60)	1.0
Ex-users	217 (6.45)	9 (4.15)	1.27 (0.62–2.59)	55 (9.24)	10 (18.18)	.86 (.41–1.80)
Current user	1227 (36.49)	56 (4.56)	1.44 (1.00–2.09)	239 (40.17)	69 (28.87)	1.56 (1.05–2.32)
**Age at first intercourse**						
≥20 y	266 (7.91)	4 (1.50)	1.0	115 (19.33)	24 (20.87)	1.0
15–19 y	2673 (79.48)	97 (3.63)	3.17 (1.14–8.77)	393 (66.05)	100 (25.45)	1.31 (.78–2.21)
<15 y	418 (12.43)	25 (5.98)	5.80 (1.96–17.10)	67 (11.26)	17 (25.37)	1.31 (.62–2.75)
**Number of lifetime male sex partners**						
0–3	N/A	N/A	N/A	296 (49.75)	52 (17.57)	1.0
4–5	N/A	N/A	N/A	273 (45.88)	89 (32.60)	2.27 (1.54–3.37)
**Number of lifetime female sex partners**						
0–3	2243 (66.70)	70 (3.12)	1.0	146 (24.54)	32 (21.92)	1.0
4–5	1112 (33.07)	56 (5.04)	1.57 (1.10–2.26)	2 (0.34)	N/A	N/A
**Number of lifetime partners with insertive anal intercourse**						
0	N/A	N/A	N/A	70 (11.76)	18 (25.71)	1.0
1	N/A	N/A	N/A	141 (23.70)	25 (17.73)	.63 (.31–1.25)
2	N/A	N/A	N/A	131 (22.02)	38 (29.01)	1.19 (.61–2.29)
3	N/A	N/A	N/A	117 (19.66)	22 (18.80)	.67 (.33–1.37)
4	N/A	N/A	N/A	75 (12.61)	24 (32.00)	1.37 (.66–2.83)
5	N/A	N/A	N/A	34 (5.71)	13 (38.24)	1.80 (0.75–4.34)
**Number of lifetime partners with receptive anal intercourse**						
0	N/A	N/A	N/A	59 (9.92)	8 (13.56)	1.0
1	N/A	N/A	N/A	130 (21.85)	21 (16.15)	1.23 (.51–2.96)
2	N/A	N/A	N/A	147 (24.71)	40 (27.21)	2.39 (1.04–5.47)
3	N/A	N/A	N/A	108 (18.15)	30 (27.78)	2.46 (1.04–5.80)
4	N/A	N/A	N/A	78 (13.11)	24 (30.77)	2.84 (1.17–6.88)
5	N/A	N/A	N/A	46 (7.73)	17 (36.96)	3.74 (1.44–9.73)
**Number of new male partners in last 6 m**						
0	N/A	N/A	N/A	212 (35.63)	52 (24.53)	1.0
1	N/A	N/A	N/A	229 (38.49)	55 (24.02)	.97 (.63–1.50)
≥2	N/A	N/A	N/A	129 (21.68)	34 (26.36)	1.10 (0.66–1.82)
**Number of new female partners in last 6 m**						
0	2005 (59.62)	71 (3.54)	1.0	132 (22.18)	28 (21.21)	1.0
1	1089 (32.38)	42 (3.86)	1.12 (0.76–1.65)	11 (1.85)	N/A	N/A
≥2	262 (7.79)	13 (4.96)	1.49 (0.81–2.74)	3 (0.50)	N/A	N/A
**Frequency of condom use in the last 6 m**						
Always	1184 (35.21)	35 (2.96)	1.0	253 (42.52)	71 (28.06)	1.0
More than half the time	670 (19.92)	25 (3.73)	1.22 (.72–2.06)	106 (17.82)	33 (31.13)	1.16 (.71–1.90)
Less than half the time	505 (15.02)	23 (4.55)	1.49 (.87–2.55)	46 (7.73)	10 (21.74)	.71 (.34–1.5)
Never	929 (27.62)	40 (4.31)	1.44 (.91–2.29)	162 (27.23)	27 (16.67)	.51 (.31–.84)
**Frequency of lifetime condom use**						
Always	1237 (36.78)	37 (2.99)	1.0	249 (41.85)	59 (23.69)	1.0
More than half the time	1090 (32.41)	48 (4.40)	1.43 (.92–2.21)	230 (38.66)	66 (28.70)	1.30 (.86–1.96)
Less than half the time	690 (20.52)	32 (4.64)	1.49 (.92–2.43)	60 (10.08)	11 (18.33)	.73 (.35–1.49)
Never	338 (10.05)	9 (2.66)	.89 (.42–1.86)	32 (5.38)	5 (15.63)	.60 (.22–1.62)
**Circumcision**						
No	2128 (63.28)	93 (4.37)	1.0	332 (55.80)	95 (28.61)	1.0
Yes	1235 (36.72)	33 (2.67)	.60 (0.40–.90)	263 (44.20)	46 (17.49)	.53 (.35–.79)
**Serology status for individual 9vHPV types^d^**						
HPV6	81 (2.41)	16 (19.75)	1.65 (.92–2.96)	97 (16.30)	58 (59.79)	4.79 (2.96–7.74)
HPV11	21 (0.62)	8 (38.10)	4.01 (1.62–9.94)	52 (8.74)	27 (51.92)	2.63 (1.45–4.76)
HPV16	28 (0.83)	6 (21.43)	1.74 (0.69–4.40)	46 (7.73)	30 (65.22)	4.80 (2.52–9.18)
HPV18	11 (0.33)	3 (27.27)	2.25 (0.58–8.66)	38 (6.39)	21 (55.26)	2.90 (1.48–5.69)
HPV31	20 (0.59)	8 (40.00)	4.42 (1.76–11.10)	24 (4.03)	15 (62.50)	3.86 (1.64–9.09)
HPV33	15 (0.45)	4 (26.67)	2.46 (0.77–7.86)	12 (2.02)	7 (58.33)	3.08 (.96–9.92)
HPV45	3 (0.09)	2 (66.67)	12.40 (1.11–13.90)	8 (1.34)	7 (87.50)	15.5 (1.89–12.70)
HPV52	11 (0.33)	4 (36.36)	3.72 (1.07–12.90)	11 (1.85)	7 (63.64)	3.84 (1.11–13.40)
HPV58	30 (0.89)	10 (33.33)	3.49 (1.59–7.65)	18 (3.03)	11 (61.11)	3.57 (1.35–9.47)

Abbreviations: 9vHPV, 9-valent human papillomavirus; HM, heterosexual men; HPV, human papillomavirus; MSM, men who have sex with men; OR, odds ratio; PCR, polymerase chain reaction.

^a^The total count of HM or MSM randomly assigned to either the placebo or vaccine arm with valid baseline PCR results for swabs at all anogenital sites among participants having all 9vHPV types tested.

^b^Number of HM or MSM in the subcategory of risk factor.

^c^Number that is in the subcategory of risk factor with concordant 9vHPV infection at 2+ sites.

^d^Association between serostatus of individual 9vHPV types (seropositivity vs seronegative or no serology) and concordant 9vHPV infection across multiple anogenital sites.

In analyses of concordant infection across 2 or more anogenital sites among MSM (including intra-anal sites), residence in Latin America (OR, 3.0; 95% CI, 1.83–5.07) or Europe (OR, 3.78; 95% CI, 2.25–6.35) was associated with concordant 9vHPV infection relative to participants located in North America (Table 4). A higher number of lifetime male sex partners (4–5 vs 0–3; OR, 2.27; 95% CI, 1.54–3.37) was also associated with concordant 9vHPV infection, along with a higher number of lifetime partners (up to a limit of 5) with receptive anal intercourse (2 vs 0: OR, 2.39; 95% CI, 1.04–5.47; 5 vs 0: OR, 3.74; 95% CI, 1.44–9.73). In addition, seropositivity for most 9vHPV types was associated with concordant 9vHPV infection, with the highest ORs observed for HPV6 (seropositivity vs seronegative or no serology: OR, 4.79; 95% CI, 2.96–7.74), HPV16 (OR, 4.80; 95% CI, 2.52–9.18), and HPV 45 (OR, 15.50; 95% CI, 1.89–12.70). Similar to HM, current tobacco use (current vs never; OR, 1.56; 95% CI, 1.05–2.32) was associated with concordant 9vHPV infection among MSM, whereas circumcision was associated with decreased odds of concordant 9vHPV infection (yes vs no; OR, 0.53; 95% CI, .35–0.79). Similar factors were associated with concordant infection in analyses of concordant infection at 2 anogenital sites among MSM (excluding intra-anal sites); however, these analyses did not identify associations for tobacco use, number of lifetime partners with receptive anal intercourse, or circumcision (Supplementary Table 3). Furthermore, multivariable logistic regression analysis of concordant 9vHPV infections (including intra-anal sites) did not identify any factors associated with concordant infection (Supplementary Table 2).

## DISCUSSION

To our knowledge, this is the first analysis evaluating concordant 9vHPV infections across multiple anogenital sites in a large cohort of young men comprising both HM and MSM. The prevalence of HPV infection with individual 9vHPV vaccine types was higher among MSM (3%–14%) than among HM (<4%), with HPV6 and HPV16 most commonly detected in both MSM and HM. The proportions of MSM with concordant 9vHPV infection at 2 (9.1%) and 3 (8.2%) anogenital sites was higher than that of HM at 2 sites (3.7%). These findings suggest that MSM with a 9vHPV infection at 1 anogenital site can be infected at multiple anogenital sites, thus increasing their risk of developing HPV-related disease at multiple sites. Concordance in MSM remained higher than HM when analyses were restricted to common anogenital sites (penile/scrotal and perineal/perianal), indicating that the risk of concordant infection among MSM is not simply a reflection of the number of anogenital sites included in the analysis.

A phase II 4vHPV vaccine trial assessing concordance of HPV infections among MSM aged 13–26 years with HIV in the United States (*N* = 145; aged 18–26 years) reported similar prevalence of concordant 9vHPV anogenital infection at 2 sites (concordance between anal and penile/scrotal sites, 11%; concordance between perianal and penile/scrotal sites, 10%) and 3 sites (concordance between anal, perianal, and penile/scrotal sites, 9%) during the prevaccination period [5]. Furthermore, the prevalence of concordant infection between anal and perianal sites (43%) was high in this study [5]. Although concordance between specific pairs of anogenital sites at a person-level (ie, anal and perianal sites) were not quantified in our analysis, the high Cohen's kappa value (0.73) for agreement of same-type 9vHPV detection at anal and perineal/perianal sites suggests that there is high concordance between anal and perineal or perianal sites among MSM. Taken together, these findings suggest that MSM with a 9vHPV infection at an intra-anal site may be infected at multiple sites in close anatomical proximity to this region (ie, perineal and perianal sites). Additional studies are also needed to further quantify the prevalence of concordant 9vHPV infection across multiple anogenital sites among HM.

In our study, the most frequently concordant individual 9vHPV types were similar between MSM (HPV types 6, 11, 16, 18, and 45) and HM (HPV types 6, 16, and 18). Previous studies enrolling MSM also reported that HPV6 and HPV16 were commonly associated with concordant infections between multiple anogenital sites, and between oral and anal sites [5, 15]. These findings are important given that HPV types 6 and 11 are associated with the development of genital warts, and HPV types 16 and 18 are associated with the development of pre-cancer and cancer. In a systematic review and meta-analysis of published studies that evaluated anal diagnoses among men or women, HPV types 6 and 11 were detected in substantial proportions of high-grade anal lesions (22.7% and 13.3%, respectively) in men without HIV who were predominantly MSM [3]; however, the importance of HPV types 6 and 11 with regards to progression from anal intraepithelial neoplasia (AIN) 2/3 to associated anal cancer is not known. Nevertheless, HPV vaccination of MSM to protect against anogenital infection with HPV types 6 and 11 will prevent the development of anal condyloma and may reduce the burden of AIN2/3 treatment.

Among HM and MSM, the degree of type-specific concordance of 9vHPV DNA detection by anogenital site pairs using Cohen's kappa values showed fair agreement for penile/scrotal and anal site concordance (MSM), fair-to-moderate agreement for penile/scrotal and perineal/perianal site concordance (HM and MSM), and strong agreement for perineal/perianal and anal site concordance for any 9vHPV type, HPV6, and HPV16 (MSM). These findings among MSM are as expected given the close proximity of perineal/perianal and anal sites, thus confirming that the likelihood of concordance is increased between anogenital sites that are anatomically closer to each other. Among MSM with anal HPV infection, HPV types 6 and 16 are the most common low-risk and high-risk HPV types, respectively [16–18]. Therefore, MSM with concordant HPV6 and/or HPV16 infection at the perineal/perianal and anal sites are at increased risk of developing associated HPV-related perineal/perianal or anal disease. Indeed, a retrospective chart review of MSM undergoing targeted ablation of perianal high-grade squamous intraepithelial lesions observed that the presence of intra-anal high-grade squamous intraepithelial lesions was associated with recurrence of perianal lesions [19].

Previous studies of risk factors associated with concordant HPV infection focused on factors associated with concordance between the oral cavity and anogenital site only [15, 20–22]. These studies found higher concordance for persons with a greater number of lifetime or recent sex partners (≥2 vs 1), HIV infection, or current smoking status [15, 20, 23]. In the current study, circumcision was associated with decreased odds of a concordant 9vHPV infection among men, while factors associated with concordant 9vHPV infection included geographic region, current tobacco use, and factors related to sexual behavior (higher number of lifetime sex partners and younger age at first intercourse). Higher risk of concordant infection in some regions (eg, Europe, Latin America, and Africa, compared with North America) suggests differing risk of disease between different regions and across multiple anatomic sites, as well as an expected greater impact of prophylactic vaccination on disease risk among males and females in regions with high vaccination rates (eg, North America). Seropositivity for selected individual 9vHPV types were also associated with concordant 9vHPV infection among men. An association between seropositivity and concordance is expected, as individuals with persistent infection are more likely to seroconvert, and longer duration of infection increases the likelihood that infections are detected at 2 or more sites [24, 25]. With the exception of circumcision among HM, these associations were lost in multivariable logistic regression analysis.

### Strengths and Limitations

The current analysis of the V501-020 trial was based on a large sample (*N* = 3958) of participants (both HM and MSM) enrolled across different geographic regions. However, there were a few limitations to this analysis. Eligibility criteria of the V501-020 trial restricted the enrollment of participants to those with no more than 5 lifetime sex partners, which may have selected for men with a low likelihood of HPV exposure. As a result, the study population of young men from the V501-020 trial may not be representative of the general population of sexually active men. In addition, concordance between infections at anogenital and oral sites could not be quantified because oral swabs were not collected in the V501-020 trial. In previous studies focusing on concordant HPV infection across oropharyngeal and genital sites in men, the prevalence of concordance varied from 1% to 3% in the general male population [20, 21, 23, 26, 27]. For example, in a cross-sectional analysis of the United States National Health and Nutrition Examination Survey (NHANES), the prevalence of concordant infection between oral and genital sites for any 9vHPV type was 2.5% among MSM and 0.4% among HM [27]. This was lower than concordance between 2 anogenital sites reported in the current analysis (MSM, 9.1%; HM, 3.7%). However, direct comparisons with the results of our analysis were not possible due to differences in vaccination coverage. This can be attributed to the study time period of the V501-020 trial (2004–2008) in which none of the men enrolled in the study were vaccinated prior to baseline assessment of HPV prevalence and impact of herd protection was much lower [6], compared with HPV vaccination rates at the time of the NHANES survey (2013–2016) when approximately 33% of MSM aged 18–26 years were reported to have been vaccinated [27].

## CONCLUSIONS

Concordant 9vHPV infection across multiple anogenital sites was more common among MSM than HM, with genotypes associated with the development of genital warts (HPV6) and pre-cancer and cancer (HPV16) most likely to be concordant. Our results are consistent with evidence that MSM are at increased risk of HPV-related cancer across multiple anogenital sites. These findings reinforce the importance of HPV vaccination, which can prevent an initial HPV infection and potentially prevent the acquisition of infections at other anogenital sites among those with a preexisting infection. The evidence from this study indicates that there is potential benefit to vaccinating males, including those with an existing infection, thus supporting the adoption of gender-neutral vaccination programs.

## Supplementary Material

jiaf564_Supplementary_Data

## Supplementary Data

Supplementary materials are available at The Journal of Infectious Diseases online (http://jid.oxfordjournals.org/). Supplementary materials consist of data provided by the authors that are published to benefit the reader. The posted materials are not copyedited. The contents of all supplementary data are the sole responsibility of the authors. Questions or messages regarding errors should be addressed to the author.
