# Supplementary material for: Assessment of Concordant Human Papillomavirus Infection With 9-Valent Vaccine Types Across Anogenital Sites in Young Men
Source: J Infect Dis. 2025 Dec 16;233(2):e462–70. doi: 10.1093/infdis/jiaf564 (PMC13017587; doi:10.1093/infdis/jiaf564)
Supplement: jiaf564_Supplementary_Data [file jiaf564_supplementary_data.docx]

**Supplementary Index**

**Supplementary Table S1.** Nonconcordant 9vHPV Anogenital Infection Status at Baseline Among Heterosexual Men and Men Who Have Sex With Men (Including Intra-anal Sites)

| **HPV Type,** | **HM (N = 3363)** | | | **MSM (N = 595)** | | | |
| --- | --- | --- | --- | --- | --- | --- | --- |
|  | **Infection with a single HPV type at 1 site^a^  n (%)** | **Infection with multiple HPV types at 1 site^a^  n (%)** | **Infection with multiple HPV types at 2 sites^b^  n (%)** | **Infection with a single HPV type at 1 site^c^  n (%)** | **Infection with multiple HPV types at 1 site^c^  n (%)** | **Infection with multiple HPV types at 2 sites^d^  n (%)** | **Infection with multiple HPV types at 3 sites^e^  n (%)** |
| 6 | 59 (1.8) | 24 (0.7) | N/A^f^ | 26 (4.4) | 9 (1.5) | N/A | N/A^f^ |
| 11 | 7 (0.2) | 6 (0.2) | N/A | 11 (1.8) | 8 (1.3) | N/A | N/A |
| 16 | 77 (2.3) | 21 (0.6) | N/A | 31 (5.2) | 14 (2.4) | N/A | N/A |
| 18 | 30 (0.9) | 13 (0.4) | N/A | 15 (2.5) | 7 (1.2) | N/A | N/A |
| 31 | 25 (0.7) | 14 (0.4) | N/A | 16 (2.7) | 6 (1.0) | N/A | N/A |
| 33 | 9 (0.3) | 4 (0.1) | N/A | 4 (0.7) | 4 (0.7) | N/A | N/A |
| 45 | 24 (0.7) | 5 (0.1) | N/A | 20 (3.4) | 0 | N/A | N/A |
| 52 | 58 (1.7) | 19 (0.6) | N/A | 14 (2.4) | 7 (1.2) | N/A | N/A |
| 58 | 22 (0.7) | 15 (0.4) | N/A | 8 (1.3) | 3 (0.5) | N/A | N/A |
| 6/11/16/18/31/33/45/52/58 | 275 (8.2) | 54 (1.6) | 17 (0.5)^g^ | 68 (11.4) | 20 (3.4) | 27 (4.5) | 4 (0.7)^g^ |

Abbreviations: 9vHPV, 9-valent human papillomavirus; HM, heterosexual men; HPV, human papillomavirus; MSM, men who have sex with men; PCR, polymerase chain reaction.

“N” indicates the number of HM or MSM randomly assigned to either the placebo or vaccine arm with valid baseline PCR results for swabs at all anogenital sites among participants having all 9vHPV types tested. One participant in the HM group was excluded due to age <16 years.

“n” indicates the number of HM or MSM with 9vHPV infection detected at baseline.

^a^Anogenital sites for HM at any 1 site comprise penile/scrotal or perineal/perianal sites.
^b^Anogenital sites for HM at 2 sites comprise penile/scrotal and perineal/perianal sites.

^c^Anogenital sites for MSM at any 1 site comprise penile/scrotal, perineal/perianal, or intra-anal sites.

^d^Anogenital sites for MSM at any 2 sites comprise penile/scrotal, perineal/perianal, or intra-anal sites.

^e^Anogenital sites for MSM at any 3 sites comprise penile/scrotal, perineal/perianal, and intra-anal sites.

^f^For a non-concordant infection with an individual HPV type, a participant cannot have same-type infection at 2 sites as this would be considered a concordant infection.

^g^For non-concordant infections with any 9vHPV type, participants may have infection with different 9vHPV types at 2 sites. This category is not mutually-exclusive from the other categories. For example, an individual with 2 infections (HPV6 at the penile/scrotal site and HPV11 at the perineal/perianal site) would be categorized as “infection with a single HPV type at 1 site” and “infection with multiple HPV types at 2 sites”.

**Supplementary Table S2.** Multivariable Logistic Regression of Factors Associated With Concordant HPV Infection (Any 9vHPV Type) at Baseline Across Multiple Anogenital Sites Among Heterosexual Men and Men Who Have Sex With Men (Including Intra-anal Sites)

|  | **HM (N = 447)^a^** | **MSM (N = 227)^a^** |
| --- | --- | --- |
|  | **Age-Adjusted OR (95% CI) of Concordant 9vHPV Infection at 2 Sites^b^** | **Age-Adjusted OR (95% CI) of Concordant 9vHPV Infection at ≥2 Sites^b^** |
| **Age^c^** | | |
| 21–27 years | 1 | 1 |
| 16–20 years | 0.88 (0.56–1.39) | 1.26 (0.62–2.55) |
| **Geographic region** | | |
| North America | 1 | 1 |
| Latin America | 1.11 (0.54–2.28) | 1.38 (0.51–3.78) |
| Europe | 1.85 (0.72–4.76) | 2.04 (0.76–5.45) |
| Asia-Pacific | 0.63 (0.12–3.30) | 0.92 (0.34–2.48) |
| Africa | 1.11 (0.54–2.31) | N/A |
| **Tobacco use at baseline** | | |
| Never | 1 | 1 |
| Ex-user | 1.83 (0.74–4.56) | 0.77 (0.25–2.42) |
| Current user | 1.22 (0.77–1.93) | 0.71 (0.38–1.34) |
| **Age at first intercourse** | | |
| ≥20 years | 1 | 1 |
| 15–19 years | 2.33 (0.71–7.59) | 0.57 (0.23–1.40) |
| <15 years | 2.96 (0.79–11.06) | 0.54 (0.15–1.89) |
| **Number of lifetime male sex partners** | | |
| 0–3 | N/A | 1 |
| 4–5 | N/A | 1.88 (0.91–3.90) |
| **Number of lifetime female sex partners** | | |
| 0–3 | 1 | N/A |
| 4–5 | 0.61 (0.37–1.00) | N/A |
| **Number of lifetime partners with insertive anal intercourse** | | |
| 0 | N/A | 1 |
| 1 | N/A | 0.74 (0.23–2.36) |
| 2 | N/A | 1.21 (0.40–3.68) |
| 3–6 | N/A | 0.59 (0.21–1.65) |
| **Number of lifetime partners with receptive anal intercourse** | | |
| 0 | N/A | 1 |
| 1 | N/A | 1.60 (0.46–5.49) |
| 2 | N/A | 2.51 (0.78–8.09) |
| 3–6 | N/A | 2.54 (0.80–8.05) |
| **Number of new male partners in last 6 months** | | |
| 0 | N/A | 1 |
| 1 | N/A | 0.51 (0.24–1.09) |
| ≥2 | N/A | 0.49 (0.19–1.30) |
| **Number of new female partners in last 6 months** | | |
| 0 | 1 | N/A |
| 1 | 1.22 (0.72–2.06) | N/A |
| ≥2 | 1.03 (0.46–2.29) | N/A |
| **Frequency of condom use in the last 6 months** | | |
| Always | 1 | 1 |
| More than half the time | 1.11 (0.49–2.53) | 1.09 (0.45–2.68) |
| Less than half the time | 1.48 (0.63–3.45) | 1.73 (0.46–6.46) |
| Never | 2.0 (0.96–4.17) | 0.74 (0.29–1.92) |
| **Frequency of lifetime condom use** | | |
| Always | 1 | 1 |
| More than half the time | 0.78 (0.37–1.65) | 0.65 (0.29–1.44) |
| Less than half the time | 0.62 (0.27–1.45) | 0.60 (0.17–2.13) |
| Never | 0.50 (0.16–1.56) | 1.17 (0.20–6.80) |
| **Circumcision** | | |
| No | 1 | 1 |
| Yes | 0.49 (0.28–0.85) | 0.94 (0.43-2.03) |

Abbreviations: HM, heterosexual men; HPV, human papillomavirus; MSM, men who have sex with men; OR, odds ratio; PCR, polymerase chain reaction.

^a^The number of observations of HM or MSM randomly assigned to either the placebo or vaccine arm with valid baseline PCR results for swabs at all anogenital sites among participants having all 9vHPV types tested in the model.

^b^The dependent variable was concordant 9vHPV infection (Yes/No). The reference level for logistic regression was nonconcordant infection at any 1 site. Individuals with no infection at any site were excluded.

^c^HM age only up to 24 years.

**Supplementary Table S3.** Factors Associated With Concordant HPV Infection (Any 9vHPV Type) Across Multiple Anogenital Sites Among Heterosexual Men and Men Who Have Sex With Men (Excluding Intra-anal Sites)

|  | **HM (N = 3363)^a^** | | | **MSM (N = 595)^a^** | | |
| --- | --- | --- | --- | --- | --- | --- |
|  | **n^b^ (%)** | **Concordant 9vHPV Infection at 2 Sites, n^c^ (%)** | **Age-Adjusted OR (95% CI)** | **n^b^ (%)** | **Concordant 9vHPV Infection at 2 Sites, n^c^ (%)** | **Age-Adjusted OR (95% CI)** |
| **Age** | | | | | | |
| 21–27 years | 1508 (44.84) | 62 (4.11) | 1.0 | 421 (70.76) | 36 (8.55) | 1.0 |
| 16–20 years | 1855 (55.16) | 64 (3.45) | 0.83 (0.58–1.19) | 174 (29.24) | 18 (10.34) | 1.23 (0.68–2.24) |
| **Geographic region** | | | | | | |
| North America | 790 (23.49) | 18 (2.28) | 1.0 | 256 (43.03) | 12 (4.69) | 1.0 |
| Latin America | 1400 (41.63) | 56 (4.00) | 1.75 (1.02–3.01) | 132 (22.18) | 20 (15.15) | 3.62 (1.71–7.67) |
| Europe | 366 (10.88) | 18 (4.92) | 2.19 (1.12–4.26) | 122 (20.50) | 18 (14.75) | 3.91 (1.79–8.54) |
| Asia-Pacific | 271 (8.06) | 2 (0.74) | 0.29 (0.07–1.26) | 85 (14.29) | 4 (4.71) | 1.03 (0.32–3.30) |
| Africa | 536 (15.94) | 32 (5.97) | 2.55 (1.41–4.60) | N/A | N/A | N/A |
| **Tobacco use at baseline** | | | | | | |
| Never used | 1919 (57.06) | 61 (3.18) | 1.0 | 301 (50.59) | 22 (7.31) | 1.0 |
| Ex-users | 217 (6.45) | 9 (4.15) | 1.27 (0.62–2.59) | 55 (9.24) | 5 (9.09) | 1.32 (0.48–3.66) |
| Current user | 1227 (36.49) | 56 (4.56) | 1.44 (1.00–2.09) | 239 (40.17) | 27 (11.30) | 1.63 (0.90–2.95) |
| **Age at first intercourse** | | | | | | |
| ≥20 years | 266 (7.91) | 4 (1.50) | 1.0 | 115 (19.33) | 8 (6.96) | 1.0 |
| 15–19 years | 2673 (79.48) | 97 (3.63) | 3.17 (1.14–8.77) | 393 (66.05) | 37 (9.41) | 1.24 (0.54–2.84) |
| <15 years | 418 (12.43) | 25 (5.98) | 5.80 (1.96–17.10) | 67 (11.26) | 9 (13.43) | 1.76 (0.61–5.07) |
| **Number of lifetime male sex partners** | | | | | | |
| 0–3 | N/A | N/A | N/A | 296 (49.75) | 18 (6.08) | 1.0 |
| 4–5 | N/A | N/A | N/A | 273 (45.88) | 36 (13.19) | 2.39 (1.32–4.32) |
| **Number of lifetime female sex partners** | | | | | | |
| 0–3 | 2243 (66.70) | 70 (3.12) | 1.0 | 146 (24.54) | 10 (6.85) | 1.0 |
| 4–5 | 1112 (33.07) | 56 (5.04) | 1.57 (1.10–2.26) | 2 (0.34) | N/A | N/A |
| **Number of lifetime partners with insertive anal intercourse** | | | | | | |
| 0 | N/A | N/A | N/A | 70 (11.76) | 10 (14.28) | 1.0 |
| 1 | N/A | N/A | N/A | 141 (23.70) | 10 (7.09) | 0.48 (0.19–1.23) |
| 2 | N/A | N/A | N/A | 131 (22.02) | 12 (9.16) | 0.64 (0.26–1.59) |
| 3 | N/A | N/A | N/A | 117 (19.66) | 7 (5.98) | 0.41 (0.15–1.14) |
| 4 | N/A | N/A | N/A | 75 (12.61) | 7 (9.33) | 0.67 (0.24–1.87) |
| 5 | N/A | N/A | N/A | 34 (5.71) | 7 (20.59) | 1.71 (0.58–5.04) |
| **Number of lifetime partners with receptive anal intercourse** | | | | | | |
| 0 | N/A | N/A | N/A | 59 (9.92) | 5 (8.47) | 1.0 |
| 1 | N/A | N/A | N/A | 130 (21.85) | 7 (5.38) | 0.62 (0.19–2.05) |
| 2 | N/A | N/A | N/A | 147 (24.71) | 17 (11.56) | 1.44 (0.51–4.12) |
| 3 | N/A | N/A | N/A | 108 (18.15) | 12 (11.11) | 1.42 (0.47–4.27) |
| 4 | N/A | N/A | N/A | 78 (13.11) | 5 (6.41) | 0.75 (0.21–2.71) |
| 5 | N/A | N/A | N/A | 46 (7.73) | 7 (15.22) | 1.97 (0.58–6.70) |
| **Number of new male partners in last 6 months** | | | | | | |
| 0 | N/A | N/A | N/A | 212 (35.63) | 19 (8.96) | 1.0 |
| 1 | N/A | N/A | N/A | 229 (38.49) | 19 (8.30) | 0.91 (0.47–1.78) |
| ≥2 | N/A | N/A | N/A | 129 (21.68) | 16 (12.40) | 1.37 (0.68–2.79) |
| **Number of new female partners in last 6 months** | | | | | | |
| 0 | 2005 (59.62) | 71 (3.54) | 1.0 | 132 (22.18) | 9 (6.82) | 1.0 |
| 1 | 1089 (32.38) | 42 (3.86) | 1.12 (0.76–1.65) | 11 (1.85) | N/A | N/A |
| ≥2 | 262 (7.79) | 13 (4.96) | 1.49 (0.81–2.74) | 3 (0.50) | N/A | N/A |
| **Frequency of condom use in the last 6 months** | | | | | | |
| Always | 1184 (35.21) | 35 (2.96) | 1.0 | 253 (42.52) | 27 (10.67) | 1.0 |
| More than half the time | 670 (19.92) | 25 (3.73) | 1.22 (0.72–2.06) | 106 (17.82) | 11 (10.37) | 0.96 (0.46–2.03) |
| Less than half the time | 505 (15.02) | 23 (4.55) | 1.49 (0.87–2.55) | 46 (7.73) | 4 (8.70) | 0.78 (0.26–2.34) |
| Never | 929 (27.62) | 40 (4.31) | 1.44 (0.91–2.29) | 162 (27.23) | 12 (7.41) | 0.68 (0.33–1.39) |
| **Frequency of lifetime condom use** | | | | | | |
| Always | 1237 (36.78) | 37 (2.99) | 1.0 | 249 (41.85) | 24 (9.64) | 1.0 |
| More than half the time | 1090 (32.41) | 48 (4.40) | 1.43 (0.92–2.21) | 230 (38.66) | 23 (10.00) | 1.08 (0.59–1.97) |
| Less than half the time | 690 (20.52) | 32 (4.64) | 1.49 (0.92–2.43) | 60 (10.08) | 4 (6.67) | 0.69 (0.23–2.08) |
| Never | 338 (10.05) | 9 (2.66) | 0.89 (0.42–1.86) | 32 (5.38) | 3 (9.38) | 0.96 (0.27–3.41) |
| **Circumcision** | | | | | | |
| No | 2128 (63.28) | 93 (4.37) | 1.0 | 332 (55.80) | 36 (10.84) | 1.0 |
| Yes | 1235 (36.72) | 33 (2.67) | 0.60 (0.40–0.90) | 263 (44.20) | 18 (6.84) | 0.61 (0.34-1.10) |
| **Serology status for individual 9vHPV types** | | | | | | |
| HPV6 | 81 (2.41) | 16 (19.75) | 1.65 (0.92–2.96) | 97 (16.30) | 19 (19.59) | 2.30 (1.24–4.28) |
| HPV11 | 21 (0.62) | 8 (38.10) | 4.01 (1.62–9.94) | 52 (8.74) | 13 (25.00) | 3.10 (1.50–6.38) |
| HPV16 | 28 (0.83) | 6 (21.43) | 1.74 (0.69–4.40) | 46 (7.73) | 11 (23.91) | 2.68 (1.26–5.69) |
| HPV18 | 11 (0.33) | 3 (27.27) | 2.25 (0.58–8.66) | 38 (6.39) | 11 (28.95) | 3.45 (1.59–7.47) |
| HPV31 | 20 (0.59) | 8 (40.00) | 4.42 (1.76–11.10) | 24 (4.03) | 3 (12.50) | 1.09 (0.31–3.79) |
| HPV33 | 15 (0.45) | 4 (26.67) | 2.46 (0.77–7.86) | 12 (2.02) | 4 (33.33) | 4.16 (1.19–14.50) |
| HPV45 | 3 (0.09) | 2 (66.67) | 12.40 (1.11–13.9) | 8 (1.34) | 5 (62.50) | 13.30 (3.06–57.7) |
| HPV52 | 11 (0.33) | 4 (36.36) | 3.72 (1.07–12.90) | 11 (1.85) | 2 (18.18) | 1.62 (0.34–7.73) |
| HPV58 | 30 (0.89) | 10 (33.33) | 3.49 (1.59–7.65) | 18 (3.03) | 4 (22.22) | 2.38 (0.74–7.63) |

Abbreviations: HM, heterosexual men; HPV, human papillomavirus; MSM, men who have sex with men; OR, odds ratio; PCR, polymerase chain reaction.

^a^The total count of HM or MSM randomly assigned to either the placebo or vaccine arm with valid baseline PCR results for swabs at all anogenital sites among participants having all 9vHPV types tested.

^b^Number of HM or MSM in the subcategory of risk factor.

^c^Number that is in the subcategory of risk factor with concordant 9vHPV infection at 2 sites.
